# Changes in Cryotolerance of Spermatozoa in Men with Teratozoospermia Under the Influence of Extracellular Vesicles from Donor Seminal Plasma Isolated by Depth Filtration

**DOI:** 10.3390/life15091436

**Published:** 2025-09-13

**Authors:** Maxim Gavrilov, Natalya Makarova, Anastasia Sysoeva, Ekaterina Evtushenko, Elizaveta Bragina, Polina Vishnyakova, Victoria Karyagina, Aida Bagdasaryan, Alexandra Yakimova, Denis Silachev, Elena Kalinina, Gennady Sukhikh

**Affiliations:** 1 Federal State Budgetary Institution “National Medical Research Center for Obstetrics, Gynecology, and Perinatology Named After Academician V.I. Kulakov”, Ministry of Health of Russia, 117997 Moscow, Russia; maxgavr67@gmail.com (M.G.); np_makarova@oparina4.ru (N.M.); sysoeva.a.p@gmail.com (A.S.); vpa2002@mail.ru (P.V.); v_karyagina@oparina4.ru (V.K.); aidabagdasaryin@gmail.com (A.B.); yakimoovaal@gmail.com (A.Y.); e_kalinina@oparina4.ru (E.K.); secretariat@oparina4.ru (G.S.); 2 N.N. Semenov Federal Research Center for Chemical Physics Russian Academy of Sciences, 119991 Moscow, Russia; 3 Faculty of Biology, Lomonosov Moscow State University, 119234 Moscow, Russia; evtushenkoea@my.msu.ru; 4 A.N. Belozersky Research Institute of Physico-Chemical Biology, Lomonosov Moscow State University, 119234 Moscow, Russia; bragor@mail.ru; 5 Research Institute of Molecular and Cellular Medicine, Peoples’ Friendship University of Russia (RUDN University), 117198 Moscow, Russia

**Keywords:** ART, seminal plasma, spermatozoa, cryopreservation

## Abstract

Currently, there is a need to improve the cryopreservation process for male gametes, especially for patients with low cryotolerance during sperm cryopreservation. Methods: The content and size of donor extracellular vesicles (EVs) in seminal plasma (SP) were assessed using nanoparticle tracking analysis (NTA), CD marker analysis, and transmission electron microscopy (TEM). Patient ejaculates were exposed to cryopreservation with or without prior co-culture with SP EVs and were not exposed to cryopreservation. The interaction of SP EVs with spermatozoa was assessed by TEM. Apoptotic, necrotic and late apoptotic cells, and mitochondrial functional activity were detected by flow cytometry. Results: NTA showed the highest concentration of SP EVs with a size of 80 nm, corresponding to small EVs. The binding of SP EVs to spermatozoa occurred along the entire plasma membrane, with an increased concentration of SP EVs at the neck and upper third of the sperm head. A significant increase in sperm motility was observed in the EVs co-culture group after cryopreservation/thawing. Flow cytometry showed a significant difference in the JC-1 Red/JC-1 Green ratio, indicating a higher mitochondrial membrane potential in the EVs exposure group. Conclusions: SP EVs have a protective function during human sperm cryopreservation.

## 1. Introduction

Male infertility contributes significantly to reproductive failure worldwide, with severe sperm morphological abnormalities (teratozoospermia) affecting a substantial proportion of infertile men and lowering fertilization rates [1]. In teratozoospermic samples, defects of the head, midpiece or tail impair motility, fertilization potential, and embryo development [1]. These morphologically abnormal spermatozoa are also more vulnerable to freezing-induced damage: cryopreservation increases the teratozoospermia index, exacerbates acrosomal and tail defects, and triggers chromatin denaturation and DNA fragmentation [2]. Thus, there is a pressing need to develop strategies that enhance the cryosurvival of sperm with severe morphological defects.

Cryopreservation of male sperm in assisted reproductive technologies (ART) infertility treatment programs is currently a routine process that allows biological material to be stored in liquid nitrogen for extended periods. Male germ cells are thought to retain their viability after thawing in 50% or more of cases [3]. However, such high cryotolerance is most often observed in sperm from donors or healthy men without somatic or other diseases. Difficulties in thawing sperm from healthy men without spermatogenesis disorders are rarely encountered in clinical practice. Challenges with sperm survival after cryopreservation arise when embryologists freeze sperm from cancer patients and testicular germ cells, for example. The low survival rate of gametes after thawing in these patients is associated with cryodamage due to thermal and osmotic shock, increased intracellular ice crystal formation, and sharply increased production of reactive oxygen species (ROS) [3]. During cryopreservation, sperm motility, viability, lipid composition, and mitochondrial membrane potential deteriorate, apoptotic pathways are activated, and fertility potential is reduced. Research is therefore continuing to improve the technology for freezing male germ cells. One approach to improve sperm survival during cryopreservation may be the use of extracellular vesicles (EVs) from seminal plasma (including donor SP).

Seminal plasma (SP) is an important component of ejaculate that plays a crucial role in the metabolism, function, survival, motility, and maturation of spermatozoa. SP consists of various macro- and microelements such as Ca^2+^, Mg^2+^, Zn^2+^, K^+^, Na^+^, and Cl^−^, which influence the fertility of male germ cells [4]. For example, Ca^2+^ is associated with sperm motility, metabolism, acrosome reaction, and fertility; Zn^2+^ is involved in antioxidant reactions affecting sperm motility; Mg^2+^ is an important cofactor in enzymatic reactions involved in energy metabolism and nucleic acid synthesis [5]. Most animal and human sperm freezing protocols include a step to remove SP by centrifugation prior to cryopreservation, which virtually minimizes the protective effect of SP antioxidant enzymes against oxidative stress.

Data obtained from animal models show that EVs isolated from follicular fluid and added during thawing can improve sperm cryotolerance [6]. Ferraz et al. demonstrated the possibility of using EVs from domestic animals of one species to successfully cryopreserve sperm samples from rare and endangered animals of another species: EVs from the oviducts of dogs and cats improved the condition of red wolf and cheetah sperm after cryopreservation. The positive effect of EVs was attributed to the fact that they can carry proteins important for sperm function, which not only improve sperm motility after thawing but also increase the preservation of the acrosome. The results of this study show that EVs can be a valuable tool for improving cryopreservation conditions for sperm from endangered species.

Another method to improve the efficiency of gamete cryopreservation is the addition of mesenchymal stem cell EVs [7,8]. Studies on the effect of adipose-derived mesenchymal stem cell conditioned medium on human sperm survival showed that total motility was 10% higher, DNA fragmentation was significantly lower, and lipid peroxidation was reduced [3].

Animal models have also demonstrated the use of SP EVs to improve gamete quality. For example, studies by Du et al. showed that boar SP EVs can enhance the antioxidant properties of sperm, reduce malondialdehyde content, maintain sperm plasma membrane integrity, improve sperm motility, and inhibit premature capacitation [9]. Fluorescence and scanning electron microscopy showed that exosomes bind directly to the sperm head membrane. The authors suggest that this improves sperm plasma membrane integrity but does not affect in vitro-induced capacitation. Numerous results from animal models provide a basis for exploring ways to increase human sperm cryotolerance in ART infertility treatment programs to improve survival and preserve fertility during freezing and subsequent thawing.

Despite these advances, there is a lack of human studies evaluating the effect of SP-derived EVs on cryotolerance in pathological sperm, and no published data on donor SP EV supplementation for teratozoospermic samples. Fertile donor EVs cargo is enriched in proteins and RNAs that are essential for sperm function and are often deficient in infertile men [10,11]. Therefore, donor SP EV supplementation may represent a novel, physiologically compatible and clinically feasible approach to improving cryosurvival in this challenging patient group.

Given the above, the aim of this study was to evaluate the role of extracellular vesicles in seminal plasma in maintaining the function and structure of human spermatozoa during standard cryopreservation, as well as to determine the feasibility and effectiveness of extracellular vesicle donation for recipients with various forms of teratozoospermia.

## 2. Materials and Methods

### 2.1. Collection of Ejaculate Samples, Seminal Plasma Isolation, and Sperm Preparation

The study was conducted at the Department of Assisted Reproductive Technologies named after Professor B.V. Leonov, Federal State Budgetary Institution “National Medical Research Center for Obstetrics, Gynecology, and Perinatology named after Academician V.I. Kulakov” of the Ministry of Health of the Russian Federation (Director: Academician of the Russian Academy of Sciences, Professor, Doctor of Medical Sciences G.T. Sukhikh, Moscow, Russia) and was approved by the Ethics Committee for Biomedical Research of the Federal State Budgetary Institution “National Medical Research Center for Obstetrics, Gynecology, and Perinatology named after Academician V.I. Kulakov” of the Ministry of Health of the Russian Federation (Protocol No. 10 dated 20 October 2022, Chairman of the Committee: Doctor of Medical Sciences D.V. Voronov). Written informed consent was obtained from each participant in the study.

Ejaculate samples were obtained from 16 patients (23–38 y.o.) with teratozoospermia and 1 sperm donor (for obtaining SP EVs) by masturbation after 2–3 days of sexual abstinence. After 30 min of liquefaction on a rotating platform, ejaculate parameters including sperm motility (%), concentration, morphology, and sperm viability were evaluated using a computer-assisted sperm analysis system (CASA, Microptic S.L., Barcelona, Spain). The donor’s parameters were as follows: total sperm concentration was 107 million/mL, progressively motile sperm was 82%, and morphologically normal sperm was 4%. This step was performed strictly according to the WHO Manual for the Examination and Processing of Human Semen (2010) [12]. Computer analysis of sperm was performed according to the manufacturer’s instructions (Microptic, Barcelona, Spain). After ejaculate analysis, density gradient centrifugation was performed to isolate the fraction of motile and morphologically normal spermatozoa in culture media according to the manufacturer’s instructions (PanECO, Moscow, Russia). A concentration gradient of 45% and 90% was used, onto which the ejaculate was layered. Next, sequential centrifugation was performed to separate the sperm fraction from the seminal plasma for 20 min at 300 g. The sediment was transferred to a test tube with a washing medium and centrifuged again for 10 min at 400 g. In this way, a fraction of spermatozoa purified from seminal plasma was obtained.

Sample size and statistical rational: Sixteen men with teratozoospermia were selected for non-parametric paired comparisons. The target sample size was determined a priori based on preliminary variability estimates obtained in an internal pilot project, which indicated approximately 10–15 percentage points difference in overall motility and viability post-thaw between treated and untreated aliquots. A two-tailed Mann–Whitney U-test (α = 0.05, power = 0.80) indicated that approximately 15–17 paired samples are required to detect such effects. Therefore, a cohort of *n* = 16 was considered statistically appropriate and logistically feasible for this pilot study.

### 2.2. Isolation of Extracellular Vesicles from Seminal Plasma by Asymmetric Depth Filtration

EVs were isolated from the SP of an official sperm donor using depth filtration cartridges according to the manufacturer’s protocol, including a cell fraction washing step followed by passage through a semi-permeable membrane (Prostagnost, Moscow, Russia). This depth filtration approach for EVs isolation has previously been described and validated in detail in [13]. Briefly, SP was separated from the cellular component of the ejaculate by centrifugation at 300× *g* for 10 min at room temperature. The supernatant was further processed in a microcentrifuge at 15,000 rpm for 30 min at room temperature. The depth filtration cartridge was prepared by introducing 2 mL of a 50% ethanol solution into the large compartment, placing the cartridge in a 50 mL tube, and centrifuging at 400× *g* for 10 min. The ethanol wets the membrane and displaces air bubbles, that interfere with the depth filtration of SP EVs. To wash away the ethanol solution, 1.5 mL of phosphate-buffered saline (PBS, pH = 7.4) (Gibco, New York, NY, USA) was added to the small compartment, and the cartridge was centrifuged at 400× *g* for 10 min. The isolated SP samples were diluted 1:10 with PBS to a final volume of 8–10 mL and introduced into the main compartment of the cartridge. The introduced sample was then centrifuged at 400× *g* for 30 min, followed by the addition of 5 mL of PBS and another centrifugation at 400× *g* for 20 min to separate proteins stuck on the membrane. To isolate the desired vesicles from the membrane, 200 µL of PBS was added to the small compartment of the cartridge and centrifuged at 400× *g* for 10 min. After isolation, SP EVs were stored at −80 °C in a laboratory freezer until use, avoiding repeated thawing.

### 2.3. Cryopreservation and Thawing of Spermatozoa

Sperm cryopreservation was performed strictly according to the manufacturer’s instructions for the SpermFreeze medium (FertiPro, Beernem, Belgium). Briefly, cryoprotectant was added dropwise to the spermatozoa in a 4:6 ratio. The cryoprotectant was mixed with the cell suspension on a rotating platform for 10 min, after which the aliquot was placed in a cryocontainer (CryoBIOSYSTEM, Santa Ana, CA, USA), which was immersed in liquid nitrogen vapor for 40 min, followed by placement in liquid nitrogen at −196 °C. Thawing was performed using SpermRINSE culture medium (Vitrolife, Gothenburg, Sweden). The cryoprotectant was removed by centrifugation at 1900 rpm for 10 min. The final aliquot volume was 1 mL.

### 2.4. Experimental Design and Co-Culturing of Spermatozoa with Seminal Plasma Extracellular Vesicles

After ejaculate processing using density gradients, each sample was divided into 3 groups: Cryo−—an aliquot without cryopreservation after washing (baseline); Cryo+—an aliquot with cryopreservation and subsequent thawing; EVsCryo+—an aliquot co-cultured with donor SP EVs, cryopreserved, and subsequently thawed. Sperm viability parameters were assessed before and after thawing for all groups. EVsCryo+ samples were co-cultured with donor SP EVs for 1 h at room temperature prior to cryopreservation. A 100 µL suspension of SP EVs was added to 300 µL of spermatozoa. The selected exosome dose was based on the minimum concentration at which a reliable effect was observed, specifically, 1 × 10^9^ vesicles. Cryo+ group spermatozoa were also kept at room temperature for the same duration under identical conditions.

### 2.5. Nanoparticle Tracking Analysis (NTA)

To assess the size and total concentration of particles in the SP EV preparation, NTA was performed using the NanoSight LM10 instrument (Malvern Instruments, Malvern, UK). Capture and analysis settings were configured according to the manufacturer’s instructions. SP EVs were visualized using laser light scattering, and the Brownian motion of EVs was recorded on video, which was analyzed using NanoSight NTA 3.1 software (Malvern, UK). The number of analyzed tracks for each video exceeded 200, and the total number of tracks for each sample exceeded 5000.

### 2.6. Western Blot Analysis of Donor Seminal Plasma Extracellular Vesicles

Extracellular vesicle markers were characterized by Western blotting. The EV sample was mixed with 2× Laemmli Sample Buffer (Bio-Rad, Hercules, CA, USA). A 10 µL sample was loaded onto a 12% polyacrylamide gel. A protein molecular weight marker (10–250 kDa, Thermo Fisher Scientific, Waltham, MA, USA) was used. Proteins were separated at 80 V for 30 min in the stacking gel and 180 V for 1 h in the separating gel. Semi-dry transfer of proteins from the gel to a PVDF membrane was performed using the Trans-Blot Turbo Transfer system (Bio-Rad, Hercules, CA, USA). After blocking non-specific antibody binding sites, the membrane was incubated with primary antibodies (Table 1): anti-CD81 (Affinity Biosciences, DF8045, 1:1000), anti-CD63 (Affinity Biosciences, AF5117, 1:500), anti-CD9 (Affinity Biosciences, AF5117, 1:1000) at +4 °C for 18 h. The membranes were then washed three times with Tris-buffered saline with 0.1% Tween-20) and incubated with Horseradish peroxidase-conjugated secondary antibodies for 1 h at room temperature. After washing, Clarity Western ECL substrate (Bio-Rad, Hercules, CA, USA) was added for chemiluminescent detection using the ChemiScope documentation system (Clinx, Shanghai, China). A densitometric analysis was performed using the ImageJ software (version 1.54b; https://imagej.net/ij/, 8 April 2025). PBS buffer without SP EVs was used as a control.

### 2.7. Transmission Electron Microscopy (TEM) of Seminal Plasma Extracellular Vesicles

For sample preparation, isolated SP EV samples were applied to 1GC300 copper grids (PELCO) coated with a collodion film and carbon coating. A 15 µL sample of SP EVs was applied to the grid. After incubation for 1 min, excess liquid was removed using filter paper, followed by negative staining with 2% aqueous uranyl acetate for 10 s and removal of excess liquid with filter paper. SP EVs were evaluated using a JEM-1400 electron microscope (JEOL, Akishima, Tokyo, Japan), a Quemesa digital camera, and iTEM 5.2 software (Olympus Soft Imaging Solutions GmbH, Münster, Germany).

### 2.8. Transmission Electron Microscopy of Spermatozoa

Spermatozoa incubated with extracellular vesicles were studied on ultrathin sections before cryopreservation. It was important to observe the interaction of EVs with spermatozoa after 1 h of incubation. Sperm samples were pre-fixed with 0.5% glutaraldehyde in isotonic NaCl solution, 2.5% glutaraldehyde in 0.1 M cacodylate buffer (pH 7.2), and osmium tetroxide, followed by dehydration and embedding in epoxy resin using standard methods. Ultrathin sections were obtained using a Reichert Jung Ultracut E microtome, mounted on copper grids coated with Formvar film, and stained with 1% aqueous uranyl acetate and lead citrate. Samples were examined at 80 kV using a JEM-1011 transmission electron microscope (JEOL, Akishima, Tokyo, Japan) equipped with an Orius^TM^ SC1000 W camera (Gatan Inc., Pleasanton, CA, USA).

### 2.9. Flow Cytometry of Spermatozoa

Detection of apoptotic cells in the samples after thawing was performed using the Annexin V-FITC kit (Abcam, Waltham, MA, USA) according to the manufacturer’s protocol. Necrotic and late apoptotic cells were detected using propidium iodide (PI) (Lumiprobe, Moscow, Russia). Measurement was performed in 500 µL of commercial binding buffer. All samples were incubated with dyes for 15 min in the dark at room temperature. Mitochondrial functional activity was analyzed using the MitoSOX Red kit (Thermo Fisher Scientific, Waltham, MA, USA) according to the manufacturer’s protocol. Measurement was performed in 300 µL of PBS. All samples were incubated with MitoSOX Red in the dark for 30 min at 37 °C. Mitochondrial membrane potential was determined using the JC-1 Mitochondrial Membrane Potential Assay Kit (Servicebio, Wuhan, China) according to the manufacturer’s protocol. Measurement was performed in 300 µL of PBS. The positive control sample was additionally treated with 0.3 µL of 100 mM carbonyl cyanide m-chlorophenyl hydrazone (CCCP), then samples were incubated at 37 °C in the dark for 30 min. Measurements were performed on a NovoCyte Advanteon flow cytometer (Agilent, Santa Clara, CA, USA). Each sample in each measurement was analyzed for 1 × 10^5^ cells. Data analysis was performed using FlowJo software v10.10: the main cell pool was gated on a dot plot of side and forward scatter, followed by exclusion of doublets and aggregates, and then the population of interest was analyzed for the desired fluorescence channels.

### 2.10. Statistical Analysis

The normality of the analyzed parameters was assessed using the Shapiro–Wilk test. Quantitative data with normal distribution were described using the mean (M) and standard deviation (±SD). The median (Me) and interquartile ranges (Q1; Q3) were used to compare ejaculate parameters. Flow cytometry data were analyzed using the nonparametric Mann–Whitney U test, with statistical significance defined as *p* < 0.05. Sample-size determination is described above.

## 3. Results

### 3.1. Native Ejaculate Characterization

At the first stage of the study, the ejaculate characteristics of the included men were evaluated. The results are shown in Table 1. The selected patients had normal sperm concentration in the ejaculate, good motility, and a reduced number of morphologically normal male gametes. Donor sperm parameters fell within normal ranges per strict Kruger morphology (WHO 2021 criteria [14]).

### 3.2. Donor’s SP EVs Characterization

The presence of donor SP EVs in the obtained aliquot and their ability to bind to spermatozoa during co-culture were analyzed using NTA, specific extracellular vesicle markers and transmission electron microscopy.

**Figure 1:**
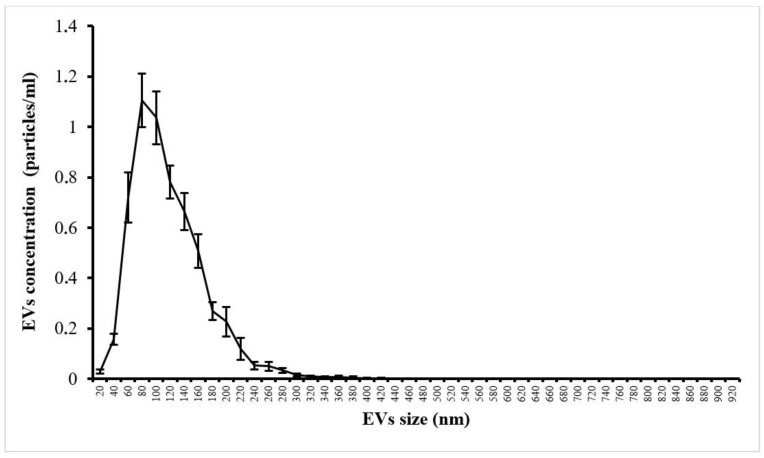
Nanoparticle tracking analysis (NTA) of extracellular vesicles from the seminal plasma of an official sperm donor. Data are presented as M ± SD.

The highest concentration of SP EVs in the donor SP preparation was observed for particles with a size of 80 nm (Figure 1).

**Figure 2:**
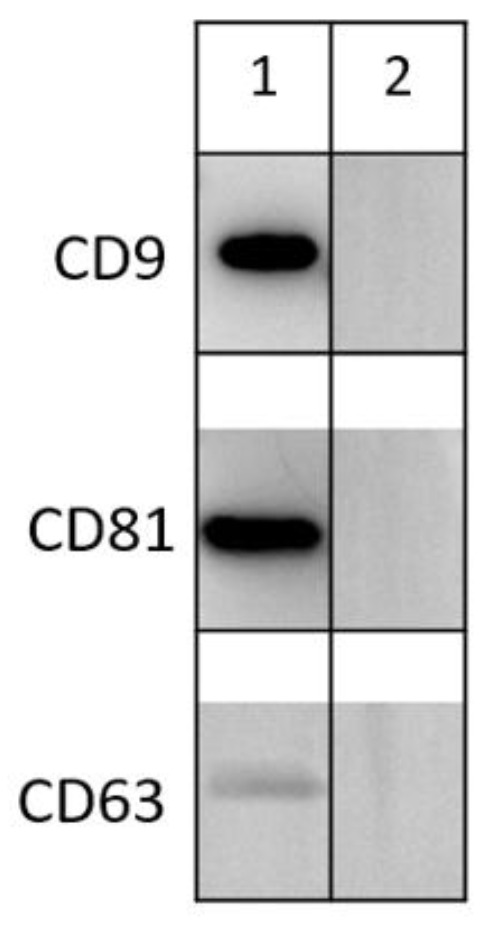
Western blot analysis of key extracellular vesicle markers (CD9, CD81, and CD63) in seminal plasma–derived EVs from a sperm donor. The observed molecular weights are 22–28 kDa. Lane 1—donor sample; Lane 2—PBS control.

Using Western blotting for membrane markers such as CD9, CD63, CD81, we demonstrated the presence of extracellular vesicles in donor SP (Figure 2).

**Figure 3:**
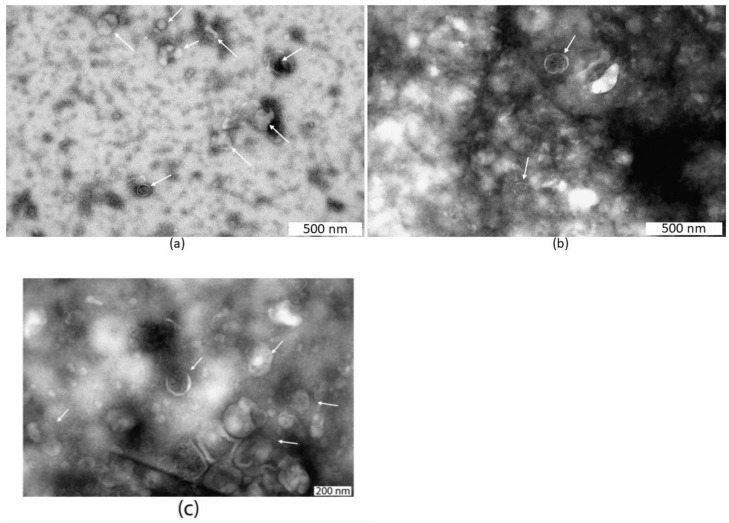
Transmission electron microscopy images of seminal plasma extracellular vesicles after negative staining with 2% aqueous uranyl acetate (**a**–**c**). Scale bars are shown on the micrographs. White arrows highlight extracellular vesicles of varying sizes present in the donor’s seminal plasma following depth filtration.

TEM analysis was used to confirm the presence of EVs in the seminal plasma sample (Figure 3). The micrographs show objects with cup-shaped morphology corresponding to SP EVs. In terms of size and characteristics, the observed vesicles can be classified as small EVs, according to MISEV2023 [15] from International Society for Extracellular Vesicles.

### 3.3. SP EVs to the Patient Spermatozoa Binding Analysis

**Figure 4:**
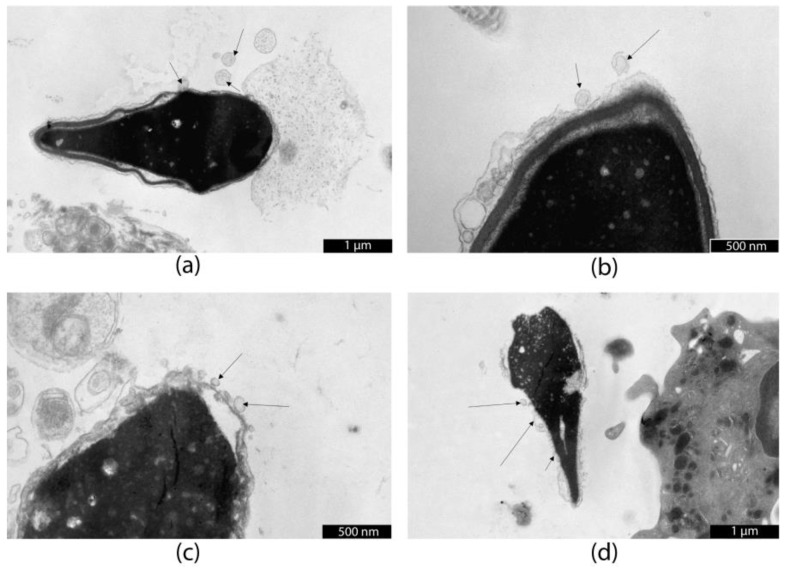
Transmission electron microscopy images of spermatozoa co-cultured with donor seminal plasma extracellular vesicles (**a**–**d**). Scale bars are shown on the micrographs. Arrows indicate SP EVs fused with the sperm cell membrane following co-incubation.

Figure 4 shows that SP EVs from donor bind to the patient’s spermatozoa in high concentrations along the entire plasma membrane. A particularly high concentration of EVs was observed at the apical part of the sperm head.

### 3.4. Evaluation of the Effectiveness of Using Donor SP EVs in Sperm Cryopreservation

#### 3.4.1. Ejaculate Characterization

The effectiveness of using donor SP EVs in sperm cryopreservation was evaluated. Ejaculate parameters after thawing are shown in Table 2. As can be seen from the data, no statistically significant difference in total sperm concentration was observed. However, the concentration of progressively motile sperm (%) was statistically significantly higher in the group co-cultured with donor SP EVs before freezing. In group 2 without SP EV preparation, motility was Me: 40 (25; 58), while in the group using SP EVs, it was Me: 47 (33; 68) (*p* < 0.05). In addition to increasing the proportion of progressively motile sperm, supplementation with donor SP EVs also improved other functional parameters after cryopreservation. Total sperm motility was significantly higher in the EVsCryo+ group than in the Cryo+ group (Me: 61 (41; 76) vs. 53 (35; 70), *p* < 0.05). Sperm viability was also better preserved after co-incubation with donor EVs prior to freezing (Me: 64 (43; 81) vs. 58.5 (42; 73), *p* < 0.05). Morphologically normal sperm (%) in all evaluated groups were Me: 3 (2;3).

#### 3.4.2. The Level of Cell Death Assessment

**Figure 5:**
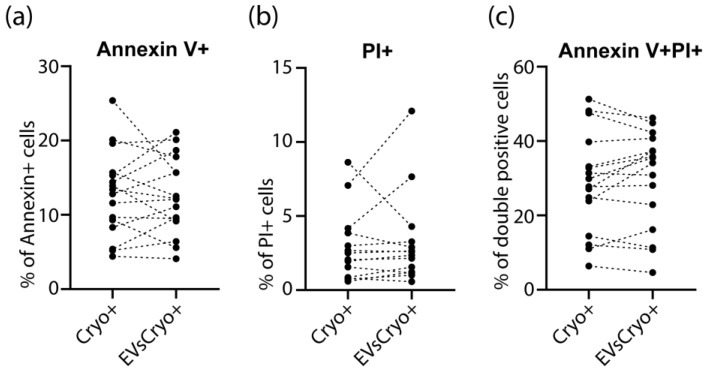
Assessment of cell death by Annexin V and PI staining. The percentages of (**a**) Annexin V-positive (early apoptosis), (**b**) PI-positive (necrosis), and (**c**) Annexin V/PI double-positive (late apoptosis) spermatozoa did not differ between the EVsCryo+ and control groups.

The percentage of Annexin V-positive cells in the early apoptotic stage did not differ between the EVsCryo+ group with the control group (Figure 5). The same observation was made for PI-positive cells. PI can only penetrate cells with permeabilized membranes, corresponding to necrotic cell death. The percentage of cells positive for both markers, reflecting late apoptosis, also did not differ between the groups.

#### 3.4.3. Measurement of Mitochondrial Membrane Potential and ROS Production

**Figure 6:**
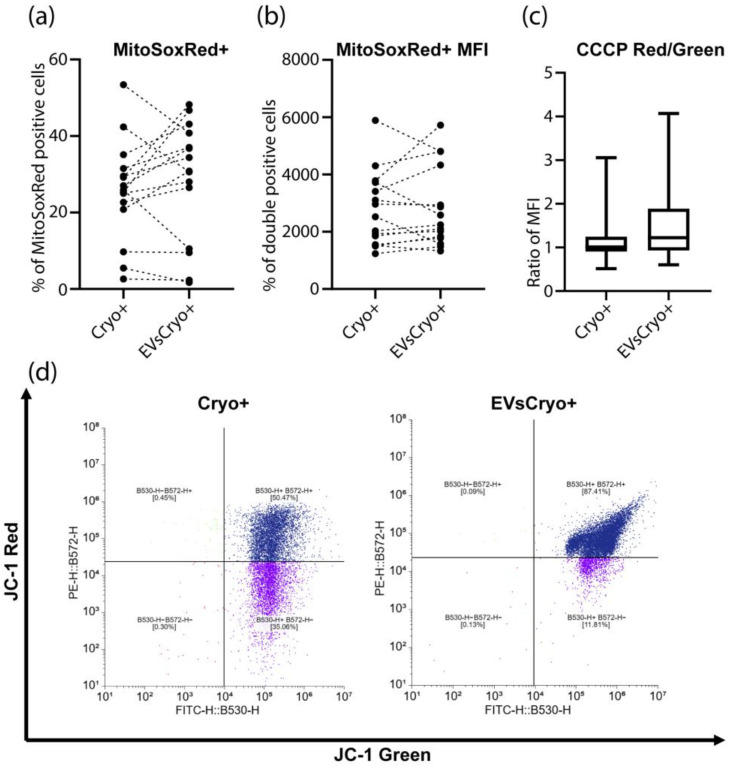
Assessment of mitochondrial function and apoptosis in spermatozoa after cryopreservation with or without EVs. (**a**) Superoxide anion production by mitochondria was evaluated using MitoSOX Red by measuring the percentage of MitoSOX Red-positive cells and (**b**) mean fluorescence intensity of MitoSOX Red staining. (**c**) Mitochondrial membrane potential was assessed using the JC-1 dye after treatment with CCCP. (**d**) Representative dot plots of JC-1 green vs. JC-1 red signal for control and vesicles treated samples. *—*p* < 0.05 by Wilcoxon test.

The production of a common mitochondrial ROS, specifically superoxide anion, by sperm mitochondria in both groups was analyzed using the MitoSOX Red dye (Figure 6). Both the percentage of MitoSOX Red-positive cells and the MitoSOX Red MFI (mean fluorescence intensity) did not differ between the groups, indicating no effect of EVs on the production of the studied ROS. Next, mitochondrial membrane potential was assessed using the JC-1 dye. As is known, in highly energized mitochondria, JC-1 fluorescence is detected in the red channel, while in mitochondria with reduced membrane potential, it is detected in the green channel. The ratio of red to green JC-1 MFI did not differ significantly between the groups.

When CCCP (carbonyl cyanide m-chlorophenyl hydrazone), a known inhibitor of mitochondrial oxidative phosphorylation that acts as a membrane potential uncoupler, was added, the EVsCryo+ group showed a significant increase in the ratio of red to green JC-1 MFI compared to the Cryo+ group, indicating a protective effect of EVs on the ability of mitochondria to maintain membrane potential despite the action of the ionophore CCCP.

The level of apoptosis in spermatozoa before and after cryopreservation did not differ significantly between the groups.

## 4. Discussion

It is known that there is a certain specificity in the binding of SP EVs to spermatozoa, depending on the binding sites. For example, Zhou et al. showed that epididymal exosomes have a specific affinity for the post-acrosomal region of the sperm head [16], while exosomes from seminal vesicles and prostate (accessory glands) have affinity for all domains of the sperm head membrane: the acrosomal ridge, acrosome, and post-acrosome [16]. Researchers suggest that exosomes binding to the sperm head may influence membrane integrity, acrosome reaction, and the ability of sperm to fuse with the oocyte, while those fusing with the midpiece and tail may have a greater impact on mitochondrial activity, energy metabolism, and motility [17,18]. In our study, an increased concentration of donor SP EVs was observed at the neck and upper third of the sperm head (Figure 4). Thus, it can be concluded that depth filtration allows the isolation of SP EVs from ejaculate in sufficient quantities, and during 1 h of co-culturing at room temperature, fusion of EVs with the membrane of male germ cells is observed. The plasma membrane is the outermost structure of the cell, acting as a barrier against foreign agents to maintain cellular structures and functions. The plasma and acrosomal membranes of spermatozoa are critically important for survival after thawing and are considered the main sites of changes after cryopreservation [19]. The structural integrity of the plasma membrane is closely related to sperm motility and viability [20]. During cryopreservation, the stability of the plasma membrane is critical for cell survival, and the fluidity and stability of the membrane determine the cryotolerance of cells, particularly spermatozoa [2,21]. It is known that the biosynthetic capacity of spermatozoa is extremely low, which is the main obstacle to self-repair when exposed to damaging agents [22]. Therefore, the function and fertility potential of male gametes largely depend on external conditions.

Our results show that SP EVs from donor not only supported the population of progressively motile spermatozoa, but also significantly improved the total motility and viability of spermatozoa after thawing. These results suggest that the protective effect of EVs goes beyond improving progressive motility, and contributes to the overall integrity and survival of male germ cells. This observation is consistent with previous animal studies that have shown that supplementation with exosomes improves sperm membrane stability, reduces oxidative stress, and increases viability after thawing [9,23]. The ability of EVs to maintain total motility and viability is particularly important in teratozoospermic samples, where cryodamage further exacerbates already compromised morphology and motility profiles. From a translational perspective, this could increase the likelihood of obtaining sufficient high-quality spermatozoa for use in ART programs, particularly in patients with teratozoospermia or other forms of male infertility characterized by low cryotolerance.

Increased ROS production and reduced antioxidant enzyme activity in spermatozoa can trigger apoptotic processes, leading to decreased sperm motility [24]. The high sensitivity of mitochondrial membranes to low temperatures can lead to ROS formation, which in turn activates the mitochondrial apoptotic pathway and electron leakage from the electron transport chain [25]. Changes in mitochondrial membrane fluidity may be another reason for ROS release [26]. In addition, mitochondrial membranes contain a large amount of polyunsaturated fatty acids, which are preferred substrates for ROS. This leads to lipid peroxidation and the formation of reactive lipid aldehydes, which covalently bind to the electron transport chain and enhance ROS formation in mitochondria. The resulting oxidative stress causes damage to the sperm plasma membrane, affecting their physiological properties, particularly the fertility profile. Damage caused by lipid peroxidation leads to reduced gamete motility. In the study [23], it was found that EVs, depending on concentration, increase antioxidant capacity and reduce lipid peroxidation damage in male germ cells during storage in liquid nitrogen, as well as preserve sperm motility.

There is evidence that extracellular vesicle composition and physical characteristics can vary considerably between individuals. Proteomic analyses of exosomes isolated from autologous blood and semen have shown donor-dependent differences in exosome concentration, size and enzymatic activity [27], and other authors have found that pooling sperm from multiple donors may reduce interpersonal variability at the cost of obscuring biologically relevant differences [28]. Although we intentionally used EVs from a single donor in our pilot study to minimize confounding and focus on proof of concept, we acknowledge that this approach limits generalisability. Future studies will examine EVs from multiple donors to determine whether donor-to-donor variation influences the protective effects observed here.

It has been reported that the seminal fluid load in men with impaired fertility- differs from the load in men with normal sperm count [29]. This heterogeneity may contribute to variability in cryotolerance and highlights the need for standardized methods to isolate and characterize EVs. In parallel, studies in animals show that supplementation with exosomes from seminal plasma improves sperm motility and prolongs effective storage time, improves the integrity of the membranes and increases antioxidant capacity while reducing lipid peroxidation during storage [9]. These results support the hypothesis that EVs provide lipids, proteins and regulatory molecules that stabilize sperm membranes, attenuate oxidative stress and prevent premature capacitation. Our findings of improved sperm quality after thawing in men with teratozoospermia are consistent with these observations and suggest that supplementation with sperm-EVs may be a valuable addition to cryopreservation media.

Mitochondria are responsible for energy production and regulate cellular metabolism, making them key components of overall cell health. Protecting mitochondria may help regulate and enhance cell viability during various in vitro manipulations, particularly cryopreservation. In the study [30], the control group without incubation with SP EVs showed significantly lower (*p* < 0.01) mitochondrial membrane potential values compared to the group in which spermatozoa were frozen after incubation with EVs [30]. In addition, the high content of polyunsaturated fatty acids in the sperm membrane and the low level of antioxidant components in the limited cytoplasm make spermatozoa more susceptible to cryodamage [31]. It has been suggested that the content of exosomes during co-culturing contributed to the protection of cells from oxidative stress, which had a positive effect on achieving redox balance in the tested groups. In groups containing 1.5 and 2.25 mg/mL of EVs, significantly higher oxidative potential values for catalase and malondialdehyde were obtained [30].

In our work, flow cytometry methods demonstrated the preservation of ATP production by sperm mitochondria after cryopreservation, which was also reflected in the number of progressively motile sperm forms (Figure 5). A high level of mitochondrial membrane potential in spermatozoa can be considered a key factor for good motility [32]. Studies by foreign authors have shown that membrane potential, which directly reflects the amount of ATP produced, is closely related to sperm functional parameters such as motility [33,34].

In this study, we used asymmetric depth filtration to isolate EVs from seminal plasma [13]. Depth filtration is a versatile and scalable method in which the sample is passed through a porous matrix with asymmetric pores. The vesicles are immobilized in the depth of the filter while smaller impurities pass through. After washing, the EVs are released by reversing the flow, resulting in a purified population. Unlike ultracentrifugation or size exclusion chromatography, this one-step approach offers high yield and purity and is suitable for large volume processing [35]. The combination of asymmetric pores and negative surface charge of the cellulose acetate prevents the adsorption of impurities to the filter and facilitates the recovery of the vesicles. As the entire thickness of the filter participates in fractionation, depth filtration is also fast, simple and cost-effective and minimizes clogging. These features make depth filtration particularly attractive for assisted reproduction technology laboratories where easy-to-use, scalable and cost-effective methods for EV isolation are required.

Several challenges must be overcome for clinical implementation. The variability of donors and the heterogeneity of the EV cargo require the development of standardized strategies for donor selection or pooling as well as strict quality control [36]. Safety risks, such as pathogen transmission, can be mitigated through strict screening protocols [37]. Determining optimal EV doses and storage conditions will be critical to maximize efficacy while avoiding adverse effects, and regulatory requirements for biologic therapeutics must be met [38].

Thus, the results of this study provide objective data supporting the results of limited animal studies, indicating that SP EVs have a protective function during human sperm cryopreservation. This may be due to both the stabilization of male germ cell membranes by EVs and the uptake of EV contents by the gametes themselves. The data obtained in this study allow for further research in this direction and the study of the proteomic, lipidomic, and genetic (especially small non-coding RNAs) composition of SP EVs to develop objective criteria for selecting SP EV donors.

## 5. Conclusions

In this study, the pre-incubation of spermatozoa with donor SP EVs showed high efficacy in preserving the population of progressively motile spermatozoa and cells with high mitochondrial potential, as determined by flow cytometry. The isolation of SP EVs by depth filtration is an accessible and simple method for any ART laboratory. The method can be popularized and disseminated to improve the efficiency of freezing male germ cells. The obtained data allow for further research in this direction, as they may help increase the efficiency of gamete cryopreservation programs in ART infertility treatment.

## Figures and Tables

**Table 1:** Parameters of native ejaculate from men included in the study, calculated using CASA.

Parameter	Value ^1^
Total sperm concentration, million/mL	79 (56; 93)
Progressively motile sperm, %	67 (59; 73)
Total sperm motility, %	78 (63; 89)
Sperm viability, %	85 (72; 95)
Morphologically normal sperm, %	3 (2; 3)

^1^ Data are presented as Me with interquartile range (Q1; Q3).

**Table 2:** Post-thaw sperm parameters with and without supplementation of donor seminal plasma extracellular vesicles (CASA analysis).

Parameter ^1^	Cryo−	Cryo+	EVsCryo+	*p* Value ^2^
Total sperm concentration, million/mL	79 (56; 93)	47 (33; 60)	34.5 (24; 45)	*p* > 0.05
Progressively motile sperm, %	67 (59; 73)	40 (25; 58)	47 (33; 68)	*p* < 0.05
Total sperm motility, %	78 (63; 89)	53 (35; 70)	61 (41; 76)	*p* < 0.05
Sperm viability, %	85 (72; 95)	58.5 (42; 73)	64 (43; 81)	*p* < 0.05

^1^ Data are presented as Me with interquartile range (Q1; Q3), n = 16; ^2^ Mann–Whitney test was used to compare Cryo+ and EVsCryo+ groups.

## Data Availability

The data presented in this study are available on request from the corresponding author.

## Abbreviations

The following abbreviations are used in this manuscript:

ART	Assisted reproductive technologies
CCCP	Carbonyl cyanide m-chlorophenyl hydrazone
EVs	Extracellular vesicles
PI	propidium iodide
PBS	Phosphate-buffered saline
ROS	Reactive oxygen species
SP	Seminal plasma
TEM	Transmission Electron Microscopy
